# Clinical Characteristics and Management of the Hydatid Cyst of the Liver: A Study from a Tertiary Care Center in Nepal

**DOI:** 10.1155/2020/8867744

**Published:** 2020-09-09

**Authors:** Utsav Joshi, Roshan Subedi, Amar Jayswal, Vishakha Agrawal

**Affiliations:** Department of GI and General Surgery, Tribhuvan University Teaching Hospital, Institute of Medicine, Tribhuvan University, Maharajgunj, Kathmandu 44600, Nepal

## Abstract

A hydatid cyst of the liver is a significant yet neglected public health problem in Nepal. The present study was carried out to evaluate the demographic characteristics, clinical presentations, and management of the patients of the hydatid cyst of the liver in the setting of a developing country. It was a retrospective, descriptive analysis of 53 patients admitted in the department of surgery with the diagnosis of hydatid cyst of the liver based on clinical manifestations, imaging studies, or serology between 2016 and 2019. The median age of the patients was 36 years, with the age group of 25-45 years being the most commonly affected (23, 43.4%). 58.5% of the patients were female. Abdominal pain (49, 92.5%) and a palpable liver (17, 32.1%) were the most common complaint and physical finding in our study population, respectively. Abdominal ultrasonography and computed tomography scan were the major imaging studies used to establish a diagnosis. A unilocular and anechoic cystic lesion was the most frequent imaging finding. The right lobe of the liver harbored the cysts in the maximum number of patients. Surgery was the preferred modality of treatment (23, 43.4%), with pericystectomy being the most common form of surgical intervention. The hydatid cyst of the liver is a common cause of morbidity in Nepal. Clinical evaluation supplemented by imaging studies is required for diagnosis, and surgery remains the treatment of choice in most cases. To explain the epidemiological pattern of the disease, multicentric studies involving a larger sample of patients should be conducted.

## 1. Introduction

A hydatid cyst of the liver (HCL), a disease most commonly caused by a tapeworm Echinococcus granulosus, is a significant yet neglected public health problem in developing nations like Nepal [1]. A World Health Organization (WHO) study in 2010 estimated the incidence of cystic echinococcosis per 100,000 population in southeast Asia to be 0.8 (95% uncertainty interval (0.2-2)) [2]. However, it is difficult to quantitate the burden of HCL in Nepal because of several reasons. First, the overall prevalence of the disease is grossly underreported in many epidemiological studies and series because systematic studies and surveys encompassing the entire endemic population have not been performed. Second, the Health Management Information System, which is the surveillance system of the Government of Nepal, tends to underreport or report the data relating to parasitic zoonosis, including HCL, incompletely [3]. Despite these shortcomings, a study by Devleesschauwer et al. showed that, between 2000 and 2012, cystic echinococcosis was only behind neurocysticercosis and congenital toxoplasmosis among parasitic zoonosis in terms of disability-adjusted life years in Nepal [1].

The life cycle of Echinococcus involves a definitive host (usually dogs), an intermediate host (such as sheep, goat, or cattle), and sometimes an incidental host (human). It is reported that almost 2-5% of domestic dogs are infected with E. granulosus in Nepal [4]. It is a common practice to slaughter domestic animals and feed raw offal to domestic dogs. The eggs released in their stool may contaminate the environment, including food, and this may be one mode of transmission to humans. The clinical manifestation of Echinococcus infection depends on the site and size of the cyst. The infection may remain asymptomatic in earlier stages when the cyst is small [5]. Eventually, as the disease progresses, HCL can present with epigastric or right upper quadrant abdominal pain, nausea, vomiting, and hepatomegaly [6].

Imaging studies, combined with immunodiagnostic techniques, often help to make a diagnosis [7, 8]. Ultrasonography (USG) is the initial imaging modality of choice because it is easy to perform, widely available, and inexpensive and can help define the number, site, size, and vitality of cysts [9]. Antibody assays can add weight to the presumptive imaging diagnosis. However, a negative serologic test rarely rules out echinococcosis [10]. Computed tomography (CT) scan and magnetic resonance imaging can help diagnose deep-seated lesions and determine the extent and condition of the avascular fluid-filled cysts [6].

Options for the treatment of HCL include surgery, percutaneous approaches, medications, and observation. Surgical removal of the cyst has remained the traditional and the gold standard for definitive management of HCL [11]. However, the alternative modalities of treatment like cyst puncture, aspiration, injection of chemicals, and reaspiration (PAIR) have progressively supplemented and in some cases even replaced surgery as the treatment of choice [6, 12]. Chemotherapy with albendazole reduces the rate of recurrence and is commonly used to complement the treatment [4].

In this study, we analyzed the demographic characteristics, clinical presentations, and management of HCL, from a tertiary care center in Nepal. This study is aimed at providing clinicians useful information about the epidemiology and clinical picture of the disease in the setting of a developing country.

## 2. Materials and Methods

This study is a retrospective, descriptive analysis of patients with HCL conducted at Tribhuvan University Teaching Hospital, a tertiary care center in Kathmandu, Nepal. Cases with the diagnosis of a hydatid cyst were identified using the nurses' admission record (January 2016 to December 2019) at the department of surgery. All available medical records of those patients were obtained and screened by researchers from January 1 to 31, 2020. The patients who had extrahepatic hydatid cysts without any involvement of the liver were excluded from the study. Out of 65 medical records screened, 53 were of the patients with HCL and were included for analysis. All the relevant data from the included patients were collected in a standard proforma. The variables used for data collection were demographic data (age and gender), clinical presentation (history and physical examination findings), laboratory investigations (hematological parameters, liver function tests, and serology), results of imaging studies (USG and CT scan), and treatment modalities.

The diagnosis was established by imaging studies and serology using an enzyme-linked immunosorbent assay (ELISA). USG and CT scan, either alone or in combination, were performed to identify and evaluate the presence of hydatid cysts in the hepatic and extrahepatic locations. The USG and CT appearance of HCL was classified based on the WHO classification system, which categorizes the cysts based on type.

The treatment strategies used in our patient population were observation, albendazole therapy, PAIR technique, modified catheterization technique, and surgery. The treatment modality was determined by the surgeons based on the type and size of the cyst.

Data was evaluated for completeness, coded, entered, and analyzed with Statistical Package for the Social Sciences, version 26. Frequencies and percentages were used to describe the categorical variables, while the mean or the median were used to describe the continuous variables.

## 3. Results

### 3.1. Demographic Characteristics

Medical records of 53 diagnosed cases of HCL admitted to the department of surgery at Tribhuvan University Teaching Hospital were obtained. The age of patients ranged from 6 years to 82 years, with a median age of 36 years. The most commonly affected age group was 25 to 45 years of age (43.4%). Among 53 patients, 58.5% were female, and the female to male ratio was 1.4 : 1. The demographic characteristics of the study population are presented in Table 1.

### 3.2. Clinical Presentation

The median duration of presentation to the hospital after the onset of symptoms was 75 days. Most of the patients presented with a complaint of abdominal pain (49, 92.5%), followed by fever (16, 30.2%). Other reported symptoms were abdominal mass, jaundice, nausea/vomiting, malaise, abdominal discomfort, and weight loss. However, three patients (5.7%) were asymptomatic at presentation and were diagnosed incidentally. The most common physical finding in our study population was a palpable liver (17, 32.1%), followed by abdominal tenderness (11, 20.8%). The clinical features are tabulated in Table 2. Thirteen patients (24.5%) had a prior history of HCL, of which three patients had been managed medically with albendazole, three had undergone PAIR, and seven had undergone surgery.

### 3.3. Investigations

The mean hemoglobin level in the study population was 12.3 gram% (SD = 1.9). Leukocytosis (leukocyte count > 11,000/cubic millimeters) was found in 20.8% of the patients, with a white blood cell count as high as 21,200/cubic millimeters. Eosinophilia (absolute eosinophil count > 500/cubic millimeters) was observed in eight patients, with a maximum absolute eosinophil count of 6201/cubic millimeters. Six patients from our study population had thrombocytopenia (platelet count < 150,000/microliter), and the other six had thrombocytosis (platelet count > 500,000/microliter). The results of the liver function tests were available for 49 of the 53 patients. 77.4% had a normal total bilirubin level (3-21 micromoles/liter), and 73.5% had a normal direct bilirubin level (0-5 micromoles/liter). Liver enzymes alanine transaminase (ALT, normal = 5-45 units/liter), aspartate transaminase (AST, normal = 5-40 units/liter), and alkaline phosphatase (ALP, normal < 306 units/liter) levels were raised in 26.4%, 24.5%, and 35.8%, respectively. Serologic evaluation using ELISA had been carried out in only 31 patients, of which 15 patients (48.4%) tested positive.

USG and CT scans were the primary imaging modalities used to make a diagnosis of HCL in our study population. Abdominal USG was done in 39 patients (73.5%), and 41 patients were investigated with an abdominal CT scan (77.3%). The maximum dimension of the cyst in USG was 12.7 cm, whereas the minimum dimension was 3 cm. Similarly, the maximum and minimum dimensions of the cyst in CT imaging were 17 cm and 2.5 cm, respectively. Thirty-six patients (70.6%) had a single hepatic hydatid cyst, whereas 15 patients (29.4%) had multiple hepatic hydatid cysts. The types of cysts observed on USG and CT scan are presented in Table 3. The right lobe was most commonly involved (78%), followed by the left lobe and the caudate lobe. The frequency of involvement of different lobes of the liver has been presented in Table 4.

Five patients with HCL had simultaneous involvement of the lungs, and four others had involvement of other organs (uterus, spleen, peritoneum, and pelvic). Of the 53 cases of HCL, 28.3% had a secondary infection of the cyst. Three patients had a dilated common bile duct or intrahepatic bile ducts; three patients had rupture of the hydatid cyst; three patients had compression of surrounding structures including gallbladder, pancreas, portal vein, and inferior vena cava; and one patient developed cystobiliary communication.

### 3.4. Treatment

Surgery was the preferred modality of treatment in our study population, as shown in Table 5. Twenty-three patients (43.4%) underwent some form of surgical intervention. The laparoscopic procedure was used in 56.5% of those cases, whereas the rest underwent open surgical intervention. Pericystectomy (14, 60.9%) was the most common form of surgical procedure in both methods. Ten patients (43.5%) underwent partial pericystectomy, while four patients (17.4%) underwent total pericystectomy. Other operative procedures that the patients underwent were combined endocystectomy and pericystectomy (4, 17.4%), endocystectomy (2, 8.7%), partial cystectomy (2, 8.7%), and exploratory laparotomy (1, 4.3%). The reason for exploratory laparotomy in one patient was cyst rupture with multiple abscesses in the abdomen. The most common indications for surgery in our study were cyst size > 10 cm, multiseptated cysts, daughter cysts, cysts with secondary infection, and concomitant extrahepatic disease. Less common indications that warranted surgery were compression of the biliary tree, gallbladder, or head of pancreas; cyst with biliary communication; and cyst rupture.

PAIR was used for the primary treatment of unilocular cysts or cysts with detached membranes, with a maximum dimension greater than 5 cm. In four patients who underwent PAIR, CT showed features of inactive cysts like heterogenous echogenicity and calcifications, whereas USG findings suggested features of active cysts, including a unilocular anechoic picture and detached membranes. Besides, the maximum dimension of the cyst was greater than 5 cm in all the four cases. Therefore, PAIR was done. Modified catheterization technique was used for the evacuation of infected cysts that were presumed to be challenging to drain via PAIR. Albendazole was used as adjunctive therapy in all the patients who were treated with PAIR, modified catheterization technique, or surgery.

Albendazole monotherapy was used as definitive therapy in cases with unilocular cysts or cysts with detached membranes in USG or CT imaging and had cysts smaller than 5 cm in greatest dimension. However, there were a few exceptions. One patient, who had a cyst size exceeding 5 cm, was managed with albendazole monotherapy. This patient underwent PAIR three months back and was given albendazole while awaiting definitive management with pericystectomy. Another case of HCL, who had multiple cysts with heterogenous echogenicity, was complicated by subdiaphragmatic, pelvic, and thoracic extension of the cyst. Despite the USG and CT picture suggesting inactive cysts, the patient was managed with albendazole for extrahepatic hydatid disease. A third patient, who had previously undergone pericystectomy with omentoplasty for an infected hydatid cyst, developed recurrent HCL. Even though the cyst had heterogenous echogenicity in USG, the patient was treated with albendazole for his recurrent disease. The other two patients, who were referred to our center, were already under albendazole before imaging done at our center showed calcified cysts.

Four patients were monitored without any treatment. Three of those patients had inactive cysts without any complications. The fourth patient, who was 82 years old, had a unilocular anechoic cyst in the liver. This patient denied albendazole administration and was kept under observation only.

## 4. Discussion

Echinococcosis remains a common public health problem in Nepal. Based on the studies done in dogs, the incidence and prevalence of Echinococcus infections are even higher in those regions where livestock rearing and slaughtering is the major source of livelihood for the people [13]. Given that the liver is the most common location of the echinococcal cysts, HCL assumes a greater importance from the public health standpoint. This study demonstrates the estimates of demographic characteristics, clinical manifestations, and management of HCL in the patients hospitalized in a tertiary care center in Nepal.

The median age of 36 years, as presented in our study, is similar to the study by Hazra et al. and Jastaniah et al. [4, 14]. Our study also shows the age group of 25-45 years as the most common age group affected by HCL. People between the ages of 25 years and 45 years are the most economically active group and comprise a significant workforce involved in agriculture and livestock rearing—which is a leading cause of HCL.

In our study, 41.5% were male and 58.5% were female, giving a male-to-female ratio of 0.70, which is in congruence with the results reported by Ahmadi and Hamidi and Abebe et al. [15, 16]. In Nepal, the female population is actively involved in household chores, agriculture, and animal husbandry. This practice is significant in rural parts of the country, where the situation is compounded by a large section of the young male population going abroad for employment opportunities, leaving behind the female population to take care of all the agricultural and livestock-related activities. This may be the reason for the female population having greater exposure to the parasite and developing HCL.

The median duration of presentation to the hospital after the onset of symptoms was 75 days in our patient population. This delayed presentation to the hospital may be because a large percentage of the at-risk population in Nepal lives in the rural parts of the country, where diagnostic and curative facilities are not available. Another reason might be the high expenses associated with seeking health care. It also reflects the low level of awareness regarding health and disease among people in Nepal.

Most of the patients included in our study presented with chief complaints of abdominal pain, followed by fever. According to Biluts et al., abdominal pain was the presenting complaint in 84% of their study population [17]. A study done in Ethiopia reported that 97.6% of the patients presented with abdominal pain. In the same study, nausea, vomiting, and weight loss accounted for a higher percentage of presenting complaints, followed by fever [16]. This is in contrast with our study in which fever was more common than nausea, vomiting, malaise, and weight loss. The higher incidence of fever in our study population is probably because of the greater number of patients with an infected hydatid cyst (28.3%). Clinical evaluation in our study population showed a palpable liver and abdominal tenderness to be the most common physical finding. This result is in line with the study by Hazra et al., which showed that 49.5% of the patients had a varying degree of hepatomegaly [4]. In almost 25% of the patients in our study, the size of the cyst exceeded 10 cm in the greatest dimension.

Nonspecific leukopenia or thrombocytopenia and nonspecific liver function abnormalities may be observed with Echinococcus infection. However, they are not diagnostic for the infection [10]. Contrary to this, our study showed leukocytosis in 20.8% of the patients, and the rest had a normal leukocyte count. Leukocytosis was most likely observed because of the secondary infection of the HCL. Platelet count was also normal in most of the patients, with 22.6% of our study population presenting with either thrombocytopenia or thrombocytosis. Almost three-quarters of the study population had a normal total and direct bilirubin, and around one-quarter had elevated liver enzyme values. Our study results are in approximation with the results of the study conducted in Ethiopia concerning leukocyte count and liver function changes [16]. All these changes in leukocyte count, platelet count, and liver function values were only seen in a fraction of the total study population and did not play a direct role in making the diagnosis of HCL, confirming their nonspecific role in the diagnosis of Echinococcus infection and HCL. Eosinophilia usually occurs if there is a rupture of the cyst and leakage of the antigenic contents [18]. In our study, just 13.2% of the patients presented with eosinophilia, which supports the notion that eosinophilia is not mandatory to diagnose Echinococcus infection or HCL.

The initial diagnosis of HCL based solely on clinical presentations may be difficult [7]. The diagnosis in our patients was established based on clinical manifestations, USG findings, CT findings, and serology. However, concurrent use of USG, CT scan, and immunodiagnostic technique to confirm the diagnosis could not be done in all the patients because of the cost issues. The sensitivity of USG for the evaluation of Echinococcus is 90 to 95% [19]. The high sensitivity combined with the low cost of USG makes it an efficient and cost-effective investigation modality in a low-income country like Nepal. In our study, the most common ultrasonographic finding was the unilocular and anechoic appearance of the cyst, followed by the heterogeneous echoic pattern within the cyst. A study by Niron et al. evaluating the USG appearance of HCL showed that 40 out of 65 cysts showed the most commonly seen features—spherical, unilocular, and anechoic—with only a few cases showing other USG findings [20]. The CT imaging in our study population also corroborates our USG findings, with the unilocular, simple cystic lesion being the most common finding. However, a greater proportion of cysts with calcified walls were detected with CT as compared to USG. This difference can be attributed to the fact that CT is superior to USG in detecting minute calcifications within the cysts, whereas USG better visualizes the active stages of the cyst [21].

We observed that the right lobe of the liver was the most common location for the hydatid cyst in our study population. Alghoury et al., in their study in Yemen, showed that 65.78% of the isolated hepatic cystic echinococcosis affected the right lobe of the liver [22]. Our study is also in agreement with the results of studies from Nepal and Greece, which present the right lobe as the most common location for HCL [4, 23]. The right lobe of the liver is affected more than the left lobe due to the nature of the portal blood flow [24]. The greater amount of blood flow may provide greater access to the oncospheres to invade the right lobe.

Antibody assays were also used in some patients to confirm the presumptive diagnosis established by imaging studies. As mentioned above, owing to the poor economic status of the patients, antibody assays could not be performed in all the patients. ELISA has a sensitivity of 56.7% to 70% but is considered highly specific for the diagnosis of human echinococcosis [23]. In our study, 28.3% of the patients had a positive ELISA result, 30.2% had a negative ELISA result, and the test could not be performed in the rest of the patients. This is in contrast with the study by Jastaniah et al. in which the serology test was positive in 25 patients (58.1%), negative in three patients (7.0%), and was not done in 15 patients (34.9%) [14]. In general, the high rate of positive serology can be attributed to the fact that the sensitivity of these serological assays is mostly evaluated in nonintact cysts, as shown by literature [25]. In a study by Aydin and Adigüzel, 48 (90.6%) out of the 53 ruptured cases tested positive, whereas only 5 (12.5%) out of the 40 cases with intact cysts tested positive in Echinococcus IgG ELISA [26]. The high rate of false-negative ELISA results in our study probably follows the same reason, given that we only had three cases of ruptured cysts in our series.

The choice of treatment is based on the types and size of the cysts, available expertise and equipment, and patients' adherence to long-term follow-up [27]. Many authors recommend surgery as the definitive modality of management, as it is the best option to remove the cysts and results in a complete cure [6, 7, 11]. In our study, 43.4% of the patients underwent surgery for definitive removal of hydatid cysts. The major form of surgery performed in our study population was partial pericystectomy. This result is in congruence with a study by Bayrak and Altıntas in which partial pericystectomy, laparoscopic, or open accounted for the significant bulk of surgeries in patients of HCL [28]. Hazra et al., in their study, found partial pericystectomy to be a tissue-sparing procedure. Also, its safety and simplicity provide an added advantage to the surgeon and the patient as compared to other surgical methods [4]. The indications for surgery in our study population closely follow those outlined by the expert meeting of the WHO-Informal Working Group on Echinococcosis in 2009. The major indications include liver cysts with daughter vesicles, superficially located cysts at risk of rupture, infected cysts when percutaneous intervention is not available, cysts with biliary communication, and cysts compressing surrounding structures [27]. In patients who did not require or qualify for surgery, PAIR, modified catheterization technique, and albendazole monotherapy were instituted as less invasive forms of management. They can be excellent alternatives to surgery in settings with limited resources and poor economic status, provided that there is no contraindication to alternative modalities of management. PAIR is mostly used for the management of >5 cm active cysts (unilocular cysts and cysts with detached membrane) [29]. Albendazole is indicated for <5 cm active cysts, patients with multiple cysts in multiple organs, peritoneal cysts, and patients who cannot undergo surgery [27, 30]. Albendazole was used as adjunctive therapy in all the patients, regardless of the modality of management. Multiple studies have confirmed the role of albendazole in the prevention of recurrence of the hydatid cyst and advocate its use as an adjuvant to standard therapies [31, 32].

One of the limitations of our study was the retrospective design. Because of the retrospective nature of the study, we could not follow up with the patients to collect the missing data in the medical records. Additional data that would have been helpful to study further variables could not be collected as well. For example, specific information regarding the exposure status of the patients to dogs or livestock and follow-up of the status of the patients after discharge from the hospital could not be evaluated in our study. We could not include the serological results of all the patients in our study, as some of them could not afford the expense of the investigation. All the cases included in our study were from a single tertiary care center and may not be representative of the features of HCL seen in the primary care setting.

Despite these limitations, the findings of this study shed light on the epidemiology of HCL in Nepal, its varied clinical picture, and the diagnostic challenges. The demographic, clinical, and therapeutic information on HCL derived from this study can be helpful to strategize clinical and public health response to the disease. Data collected from the endemic region like Nepal help to evaluate the regional burden of disease, assess the effectiveness of control programs, and shape the diagnostic and treatment guidelines [33]. Multicentric studies involving a larger sample of patients should be conducted to complement the existing knowledge on the epidemiological pattern of the disease.

## 5. Conclusion

The hydatid cyst of the liver is a significant but neglected public health problem in Nepal. The demographic, clinical, and therapeutic information on HCL derived from this study can be helpful to strategize clinical and public health response to the disease. Promoting awareness on the disease and ensuring the availability of the USG facilities in different parts of Nepal are necessary. Multicentric studies involving a larger sample of patients should be conducted to explain the epidemiological pattern of the disease.

## Figures and Tables

**Table 1:** Demographics of the study population (*n* = 53).

	Number	%
Age group (in years)		
5-25	10	18.9
25-45	23	43.4
45-65	13	24.5
>65	7	13.2
Sex		
Male	22	41.5
Female	31	58.5

**Table 2:** Clinical features in the study population.

Clinical features	Number (%)
History	
Abdominal pain	49 (92.5)
Fever	16 (30.2)
Jaundice	5 (9.4)
Abdominal mass	5 (9.4)
Nausea/vomiting	4 (7.5)
Physical examination	
Palpable liver	17 (32.1)
Abdominal tenderness	11 (20.8)
Icterus	5 (9.4)
Abdominal distension	3 (5.7)

**Table 3:** Cyst characteristics in USG and CT abdomen.

Cyst characteristics	Number	%
USG abdomen		
Unilocular anechoic cystic lesion	13	33.3
Multiseptated cyst	3	7.7
Cyst with detached membranes	5	12.8
Cyst with daughter cysts	5	12.8
Cyst with heterogenous contents	8	20.5
Calcified wall	2	5.1
Features suggestive of infection	3	7.7
Total	39	100.0
CT abdomen		
Unilocular simple cystic lesion	12	29.3
Multiseptated cyst	4	9.8
Cyst with detached membranes	5	12.2
Cyst with daughter cysts	5	12.2
Cyst with heterogenous contents	1	2.4
Calcified wall	11	26.8
Features suggestive of infection	3	7.3
Total	41	100.0

**Table 4:** Involvement of different lobes of the liver (*n* = 50).

Lobe of liver	Number (%)
Right lobe only	29 (58)
Left lobe only	9 (18)
Caudate lobe only	1 (2)
Right and left lobes	9 (18)
Right and caudate lobes	1 (2)
All lobes	1 (2)

**Table 5:** Modalities of treatment.

Modalities of treatment	Number	%
Observation	4	7.5
Albendazole monotherapy	9	17.0
PAIR (+albendazole)	13	24.5
Surgery (+albendazole)	23	43.4
Modified catheterization techniques (+albendazole)	4	7.6

## Data Availability

The authors confirm that the data supporting the findings of this study are available within the article.
